# Integrating anatomical and functional landmarks for interparticipant alignment of imaging data

**DOI:** 10.1162/imag_a_00253

**Published:** 2024-08-01

**Authors:** Jayson Jeganathan, Bryan Paton, Nikitas Koussis, Michael Breakspear

**Affiliations:** School of Psychological Sciences, College of Engineering, Science and Environment, University of Newcastle, Newcastle, NSW, Australia; Hunter Medical Research Institute, Newcastle, NSW, Australia; School of Medicine and Public Health, College of Medicine, Health and Wellbeing, University of Newcastle, Newcastle, NSW, Australia

**Keywords:** hyperalignment, functional alignment, decoding

## Abstract

Aligning brain maps using functional features rather than anatomical landmarks potentially improves individual identifiability and increases power in group neuroimaging studies. However, alignment based purely on functional magnetic resonance imaging (fMRI) risks omitting useful anatomical constraints. An optimized combination of anatomical and functional feature alignment could balance the advantages of each approach. We used 3T fMRI data from 80 Human Connectome Project participants during seven tasks. The effectiveness of functional and anatomical alignment methods was evaluated using interparticipant decoding accuracy. Functional alignment mapped vertices from participants to a template, aligning their fMRI responses to shared responses during movie viewing. The template was derived from the combined fMRI responses of a set of participants. We benchmarked the results against existing functional alignment methods, including the Procrustes method and ridge regression. A common practice in the field is to use the same participants for the alignment cohort and for template generation. We found that this inflates decoding accuracies by mixing anatomical and functional alignment. Based on this, we recommend that a template’s generalizability should be evaluated against held-out participants. Building on these findings, we investigated whether inter-subject alignment could be improved by integrating anatomical and functional information. We studied a modified alignment method where a single parameter interpolates between pure functional alignment and anatomical alignment. Optimizing the parameter with nested cross-validation, we found that integrating anatomical and functional information robustly reduced noise and improved alignment across a variety of alignment methods. Combining anatomical and functional information accounts for individual heterogeneity in functional topographies while incorporating anatomical constraints. The integrated alignment described here improves inter-subject decoding using functional brain maps. These findings also demonstrate that brain anatomy provides a lens into the inherent variability of individual neural landscapes.

## 1 Introduction

The ability to classify and distinguish participants’ behavior from brain scans is a cornerstone of MRI research and potential clinical applications. These studies usually align participants’ brains to a common template. Traditional MRI studies align participants’ brains to a template space based on anatomical landmarks (Jenkinson et al., 2002). However, there are substantial differences between individuals in the anatomical loci corresponding to particular functions (Haxby et al., 2001), with residual variability even after alignment with MSMAll (Glasser et al., 2016). Overlooking the rich functional variability across individuals may limit the success of interparticipant alignment and subsequent brain-based prediction. In this context, using functional rather than anatomical landmarks could improve interparticipant alignment, with the promise of reducing the sample sizes needed for brain-based prediction (Haxby et al., 2020).

Functional alignment, also known as hyperalignment (Haxby et al., 2011), rests on the premise that functional activations to the same stimulus in different individuals may occur in different anatomical loci. These individual differences can be characterized once all individuals’ functional activations have been aligned to a common stimulus set, for instance to the timepoints of a viewed movie. Functional alignment typically operates in pairs, finding a linear transformation from a source participant’s vertices (or voxels) to a target participant’s vertices (or voxels), transforming functional activations in the source participant to maximally overlap with activations in the target participant. This assumes that activations in one vertex in the target participant can be described by linearly combining activations in one or more vertices in the source participant. This mapping is assumed to be invariant across time and different stimuli, so that once the mapping is derived, it can be successfully used to “align” participants’ responses to new stimuli. In other words, if we are given a source participant’s functional activation map for a new stimulus, a functional alignment map will predict a target participant’s functional activation to that stimulus. In large group studies, the functional activation maps of all participants are mapped to a common “template” participant. Functional alignment improves the similarity between participants’ functional activation profiles compared to anatomical registration alone (Bazeille et al., 2019,^2021^;Guntupalli et al., 2018;Haxby et al., 2020) and improves prediction of cognitive phenotypes (Feilong et al., 2021). Functional alignment refers to non-diffeomorphic alignments calculated from purely functional data, and differs from diffeomorphic approaches like multimodal surface matching (MSM) (Robinson et al., 2014). MSM calculates a smooth, invertible, and topology-preserving transformation between participants’ meshes based on structural and/or functional features. In contrast, functional alignment eschews these constraints, permitting a wider range of possible transformations.

In functional alignment, template alignment consists of two steps: (1) template generation and (2) aligning participants to the template. In most previous work, the same participants were used for template generation and for alignment (Bazeille et al., 2019;Feilong et al., 2021;Guntupalli et al., 2018;Haxby et al., 2011). That is, the participants aligned to the template in step 2 are the same participants from whose data the template is derived. The utility of a template relies on its generalizability beyond those participants from whose data it was derived. Therefore, we tested whether generating the template with an out-of-sample set of participants impacted the success of functional alignment. The results of this exploration unexpectedly led to the conclusion that retaining potentially useful anatomical constraints can improve on functional alignment. We developed “integrated alignment,” a framework that interpolates between pure anatomical alignment and pure functional alignment, balancing the advantages of both approaches. Integrated alignment robustly improved the concordance between participants’ brain maps compared to either anatomical or functional alignment alone. We benchmark the utility and performance of our approach against methods such as the ProMises model, which pursues a similar outcome using spatial regularization (Andreella et al., 2022).

## 2 Methods

### 2.1 Data

We used minimally preprocessed Human Connectome Project data from the first 80 participants who had completed the required scans. These were 7T movie viewing fMRI, and 3T fMRI scans for seven tasks: working memory, gambling, relational, motor, emotion, language, and social (Andersson et al., 2003;Andersson & Sotiropoulos, 2015,^2016^;Feinberg et al., 2010;Fischl, 2012;Glasser et al., 2013;Glasser & Van Essen, 2011;Jenkinson et al., 2002,^2012^;Milchenko & Marcus, 2013;Moeller et al., 2010;Robinson et al., 2014,^2018^;Setsompop et al., 2012;Sotiropoulos et al., 2013;Van Essen et al., 2012;Woolrich et al., 2001). Openly available de-identified data from the project were used in accordance with the HCP Data Use Agreement. This study was approved by the University of Newcastle Human Research Ethics Committee (H-2020-0443).

**Fig. 1.**
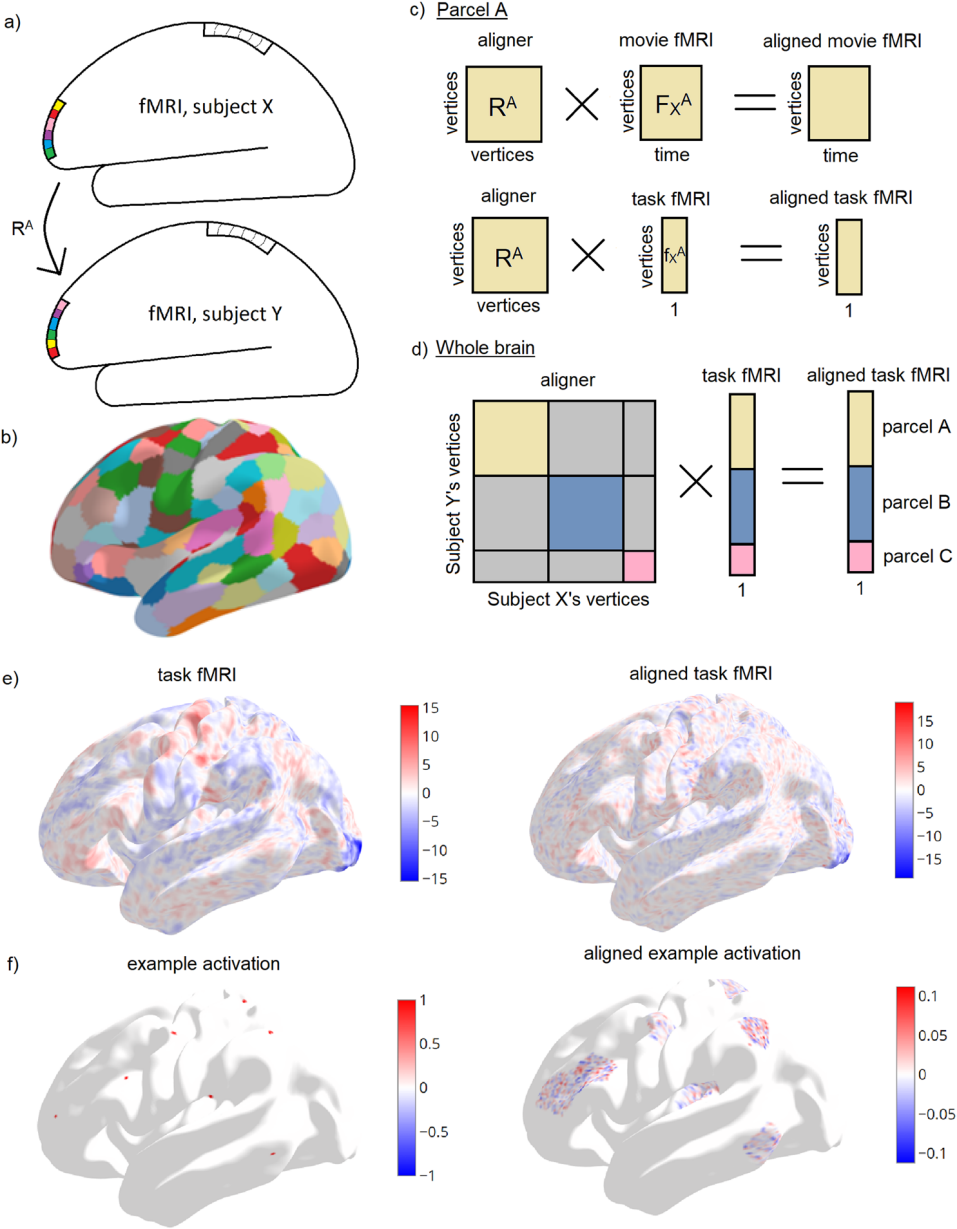
Functional alignment methodology. (a) Functional alignment accommodates rearrangements in functional topography across individuals. Boxes represent vertices within a parcel. In this example, each parcel contains six vertices. Colors represent different functional roles. (b) 300 region cortical parcellation (Schaefer et al., 2018) used throughout this paper. (c) Alignment matrix R^A^maps from vertices in parcel A in participant X to vertices in parcel A in participant Y, effectively matching vertices with similar fMRI responses to movie viewing. Alignment transformations {R^A^, R^B^,…} were calculated for each parcel {A, B,…} separately. The transformation generalizes to different functional modalities. The transformation, when applied to participant X’s task fMRI response, predicts participant Y’s task fMRI response. (d) Per-parcel alignment matrices {R^A^, R^B^,…} were concatenated into a block diagonal whole-brain transformation R^full^. All elements in off-diagonal grey blocks are zero. (e) A single participant’s fMRI map for “right hand” motor task before (left) and after (right) transformation to another participant’s (movie-derived) functional space. (f) A toy example of localized region-of-interest (ROI) activations before (left) and after (right) transformation to another participant’s functional space, showing that functional activations remain within their original parcel after alignment.

Cortical data were aligned to the fs32kLR surface space based on anatomical features (MSMSulc). While most previous studies on hyperalignment have used anatomically aligned data, we also compared using cortical data aligned using anatomy, myelin architecture, and resting-state connectivity (MSMAll). Functional alignment is an additional non-diffeomorphic alignment step after diffeomorphic registration with MSMSulc or MSMAll. This allowed us to compare the following pipelines: MSMSulc only, MSMSulc + functional alignment, MSMAll only, and MSMAll + functional alignment. Functional alignment transformations were estimated from participants’ fMRI responses to 1 hour of movie viewing. Benefits of alignment were quantified by increased interparticipant overlap in task fMRI. For parcellation-based analyses, the fs32kLR cortical surface space was subdivided into 300 parcels using a functional parcellation with mean parcel diameter of 24 mm (Schaefer2018_300Parcels_Kong2022_17Networks) (Kong et al., 2021;Schaefer et al., 2018) (Fig. 1b).Supplementary Methods 1outlines further details of the data used.

### 2.2 Functional alignment from source to target

Following anatomical surface-based registration, vertices in source and target participants may not be in correspondence, due to poor performance of the co-registration algorithm, or because vertices with similar functional profiles may be located at different anatomical locations in different participants. Functional alignment seeks to match vertices in source and target participants by the similarity of their functional profile. Functional alignment calculates a transformation mapping from vertices in a source participant X to vertices in a target participant Y, aiming to predict functional activations in the target participant from knowledge of the source participant. We estimated these transformations using participants’ fMRI responses to a common stimulus, namely 1 hour of movie viewing.

Let$F_{X}^{v\times p}$be a matrix representing the functional response of participant X to movie-viewing, with v vertices (rows) and p timepoints (columns). We note that this is different to the notation used in some studies, where vertices are represented in columns. Functional alignment estimates matrix$R^{v\times v}$to minimize the difference between$R\times F_{X}$and$F_{Y}$, where$R\times F_{X}$denotes the functional response of source participant X transformed to the functional space of target participant Y. We used three different methods to optimize R, described inSection 2.4.

Alignment was computed separately within parcels. This constraint prevents unrealistic alignments, for instance those that map the left hemisphere of one participant to the right hemisphere of another participant. The resulting parcel-specific alignment matrices$\left\{{\text{R}}^{\text{A}},{\text{R}}^{\text{B}},{\text{R}}^{\text{C}},\ldots \right\}$were concatenated into a block diagonal whole-cortex alignment matrix${\text{R}}^{\text{full}}$(Fig. 1d).

If functional alignment improves registration above and beyond anatomical registration, it should increase the concordance between two participants’ brain activations to a common stimulus. The common stimulus tested here should be different to the stimulus used to calculate the alignment. Here, we tested movie-derived alignment against task fMRI contrast maps. Let$f_{X}^{v\times 1}$be the task fMRI contrast map of participant X with v vertices. Each parcel A in$f_{X}$was transformed by the parcel-specific aligner$R^{A}$. Equivalently, the whole-cortex task map$f_{X}$was transformed by block-diagonal whole-cortex aligner$R^{full}$(Fig. 1c, d).

$$\text{predicted}\;f_{Y}^{A}=R^{A}\times f_{X}^{A}$$

$$\text{predicted}\;f_{Y}=R^{full}\times f_{X}$$

To better understand the transformations, we generated a toy example of highly localized region-of-interest activations in a source participant. This activation map was visualized before and after alignment (Fig. 1f). To generate this example, a set of center vertices were randomly selected. All vertices within 1 mm of any center vertex were given value 1. All vertices not within 1 mm of any center vertex were given value 0.

### 2.3 Validating functional alignment

**Fig. 2.**
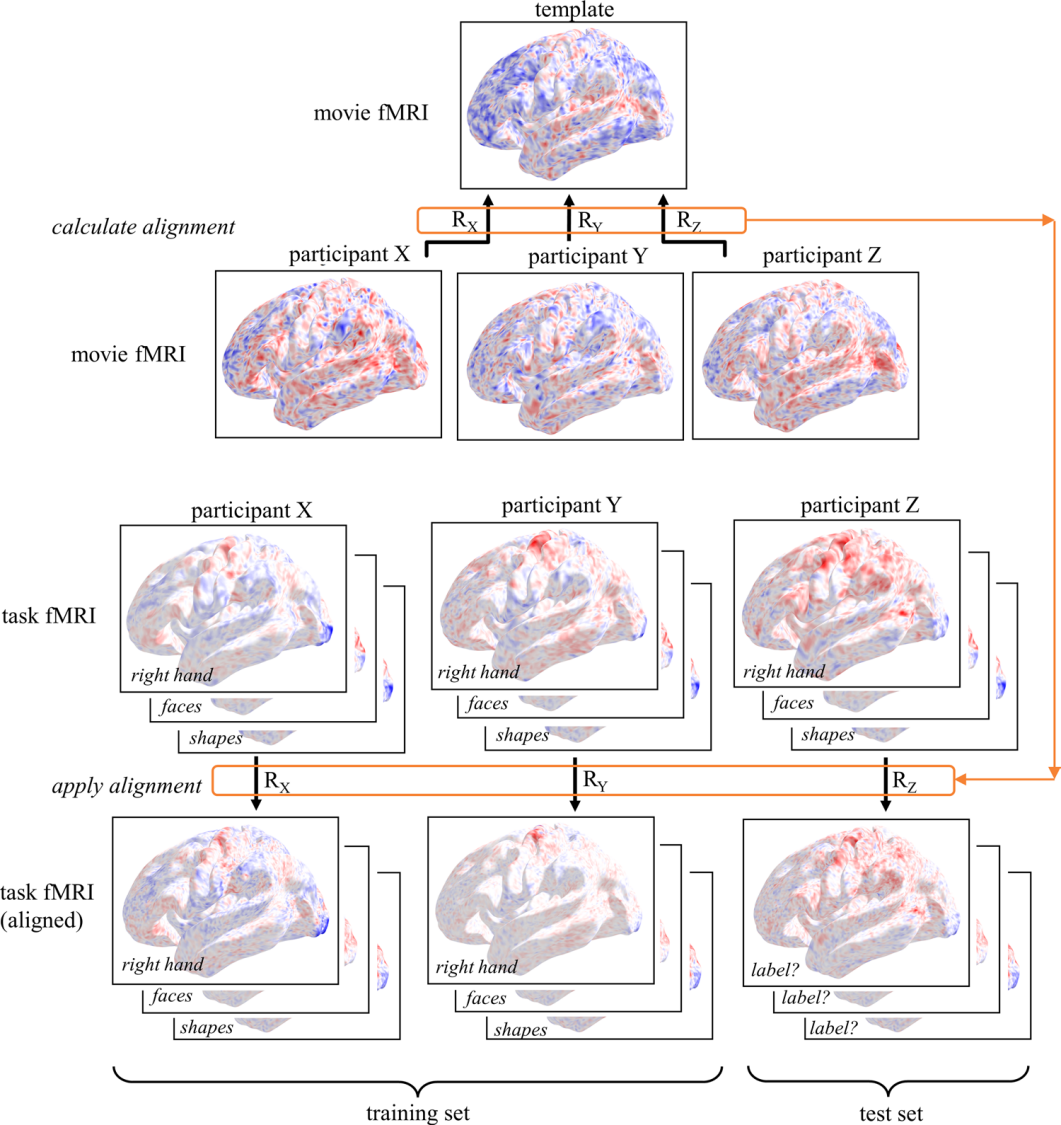
Schematic demonstrating the decoding paradigm for evaluating alignment methods. Alignment transformations were calculated from each participant to a common template, based on movie viewing fMRI responses. These participant-specific transformations were applied to each task fMRI contrast map in each participant, to align them into a common functional space. Participants were divided into training and test sets. A linear support vector classifier was trained on aligned task fMRI maps to decode task labels. The classifier was used to decode the task label of each contrast map in each participant in the test set. We compared classification accuracies when task fMRI maps were functionally aligned, versus not functionally aligned (i.e., anatomically aligned only).

The performance of functional alignment in contrast to anatomical alignment alone was quantified with an interparticipant decoding framework (Fig. 2). Compared to measuring the correlation between participants’ activations, a decoding framework is less susceptible to non-specific effects of alignment such as smoothing. Each participant had a set of 18 task fMRI contrast maps matched to 18 task labels (left hand, viewing faces, etc.). Task contrast maps, with or without functionally alignment to a common target, were used for task label classification. Participants were divided into training and test sets. A linear support vector classifier was trained on task contrast maps in the training set, to predict out-of-sample task labels. Classifiers were implemented without feature selection and with default model regularization. The classifier provided a classification accuracy score in the test set (chance level 1/18). Generalizability was evaluated with 5-fold cross-validation.

Some functional alignment methods, by remixing the values within each parcel, will reset the mean activation of every parcel to the same value (explained further inSupplementary Methods 2). If differences between parcels’ mean activations are important for classification, then by discarding this information, functional alignment will underperform. To ensure a fair comparison of functional and anatomical alignment, we appended parcel-specific means as additional features to functionally aligned task fMRI maps before training and testing the classifier.Supplementary Methods 2discusses the rationale for this in more detail.

### 2.4 Source-to-target alignment algorithms

We used three different source-to-target algorithms to estimate the parcel-specific alignment matrix R. Algorithms were implemented in the*fmralign*Python package. These algorithms estimate the transformation from a source participant X to a target Y, within a single parcel. We outline the three source-to-target algorithms below, using similar notation conventions to those introduced previously (Bazeille et al., 2021).

#### 2.4.1 Procrustes algorithm

The Procrustes algorithm constrains transformation R to be the product of a scaling factor and an orthonormal rotation matrix (Haxby et al., 2011). The orthonormal component preserves the vector norms of activation maps and preserves angles between pairs of activations maps. The algorithm finds matrix R satisfying the following equation,

$$\underset{R=sM}{min}\|\;RF_{X}-F_{Y}{\|}_{2}^{2},\;\;s\in R^{+},\;M\in R^{v\times v},\text{such that}\;M^{T}M=I_{v}$$

The first application of functional alignment to human fMRI data was termed “hyperalignment,” and used the Procrustes algorithm together with an iteratively generated template (Haxby et al., 2011).

#### 2.4.2 Optimal transport

The optimal transport algorithm estimates a transformation R that minimizes cost, and includes an entropic smoothing term (Bazeille et al., 2019). Let$F_{X}^{i}$represent the functional response of vertex i in participant X. The cost of mapping vertex i in source participant X to vertex j in target participant Y is the dissimilarity between the functional response of these vertices given by matrix$C^{v\;\times \;v}$, where$C_{i,j}=\left\|F_{X}^{i}-F_{Y}^{i}\right\|$. The total cost of the transport plan R is given bytrace(R·C). Total cost is minimized when source vertices are mapped to target vertices with similar functional profiles.

The discrete entropy of transformation R is defined as follows:

$$H\left(R\right)=-\sum_{i,j}R_{i,j}\left(log\left(R_{i,j}\right)-1\right)$$

We use the Sinkhorn algorithm to find a matrix R that minimizes for the following,

$$\underset{R\in {\mathbb{R}}_{+}^{v\times v}}{\min}trace\left(R\cdot C\right)-\epsilon H\left(R\right)$$

The user-set hyperparameterϵcontrols the sparsity of the mapping.ϵwas set to 1 throughout these experiments, as this value maximized task fMRI classification accuracy in a held-out sample. Each row and column of R sums to 1, meaning that all vertices are given equal weight.

#### 2.4.3 Ridge regression

The ridge regression algorithm used L_2_regularization to prefer sparser solutions with smaller matrix norms,

$$\underset{R}{min}{\|RF}_{X}-F_{Y}{\|}_{2}^{2}+\alpha {\left\|R\right\|}_{2}^{2}$$

User-set hyperparameter α was set to 1000 throughout these experiments, as this value maximized task fMRI classification accuracy in a held-out sample.

### 2.5 Choosing a common target

The above describes source-to-target alignment algorithms from one participant to another. In group studies, all participants need to be aligned to a common functional space. The selected source-to-target algorithm was used to calculate a mapping from each participant’s movie viewing fMRI to the movie viewing fMRI of a common template. Then, to test the alignment, each participant’s task fMRI was transformed using their respective alignment matrix. Finally, the concordance between aligned task fMRI maps was evaluated as described inSection 2.3.

#### 2.5.1 Template alignment

The common template was generated by combining data from many participants. A template constructed from many participants’ functional data is less noisy than a single participant’s functional data. Therefore, aligners mapping from one participant to a template will be less noisy than aligners mapping from one participant to another participant. Templates were generated separately for each parcel and then concatenated. Just like participants’ individual data, the final template is an$F^{v\times p}$matrix, where v is the number of vertices and p is the number of timepoints. Once the template is generated, transformations can be calculated to align any participant to that template. In previous studies, the participants who were aligned to the template were the same as the participants used for template generation. We investigated whether the template was generalizable; that is, whether one could use different participants for the two groups. We now outline three methods of generating the template: Generalized Procrustes Analysis (GPA), hyperalignment, and principal component analysis (PCA). These are described in further detail inSupplementary Methods 3.

#### 2.5.2 Template generation with generalized Procrustes analysis

The GPA method as implemented in the*fmralign*package generates a template iteratively (Gower, 1975). The initial template was the Euclidean mean of the images of participants in the template generation cohort. Each participant’s image was aligned to this template. The Euclidean mean of participants’ aligned images formed a new template. This procedure was iterated to form the final template (Supplementary Methods 3.1).

#### 2.5.3 Template generation with hyperalignment

The hyperalignment method, as implemented in the*PyMVPA2*package, comprised two “levels.” In level 1, participants were aligned sequentially to a growing template, starting with a single, arbitrarily chosen participant as the initial template (Haxby et al., 2011). After each participant was aligned to the template, a new template was defined as the Euclidean mean of the previous template and the latest participant’s aligned image. In level 2, a new template was defined as the Euclidean mean of all aligned images from level 1. Each participant’s image was aligned to this template. The final template was the Euclidean mean of all participants’ level 2 aligned images (Supplementary Methods 3.2).

#### 2.5.4 Template generation with principal component analysis

The “individualized neural tuning model” (Feilong et al., 2023) combined four components: principal component analysis (PCA) to generate a template, orthogonal rotation of this template, mapping participants’ images to the template with ridge regression, and bootstrap aggregation. Here, we used a method containing the first two of these steps, conducted for each parcel separately. Participants’ images were concatenated across the “vertices” axis. The principal components of this matrix represented linear combinations of vertices from multiple participants. A reduced set of these components, with an additional Procrustes rotation step, formed the template (Supplementary Methods 3.3).

#### 2.5.5 Pairwise alignment

Instead of aligning each participants to a template derived from multiple participants’ data, the participants could be aligned to a single target participant’s data. This was termed the “pairwise” method. Transformations were calculated from each “source” participant to the target participant’s movie viewing fMRI. These transformations were used to transform each source participant’s task fMRI to the target participant’s functional space. The classifier was trained on the source participants’ aligned task fMRI and tested on the target participant’s task fMRI. We repeated analyses using each of the participants in turn as the target participant and averaged the classification accuracies. The pairwise method is computationally expensive because it requires the estimation of transformations between all participant pairs.

#### 2.5.6 Combining source-to-target algorithms with methods for choosing a target

In total, we considered four methods of choosing a target—the pairwise method, and three methods for deriving a multi-participant template. All four methods of choosing a target are compatible with any of the three source-to-target alignment algorithms (Table 1). For example, “GPA template Procrustes” refers to using the Procrustes algorithm to align participants to a target generated with the GPA method. This combination was primarily used throughout this paper, with additional analyses using “PCA template ridge regression” and “pairwise optimal transport.”

**Table 1.** Source-to-target algorithms (columns) and methods of choosing a target (rows) used in this paper.

	Source-to-target algorithms		
Choosing a target	Procrustes	Optimal transport	Ridge
Pairwise		Pairwise optimal transport	
GPA template	GPA template Procrustes		
Hyperalignment template			
PCA template			PCA template ridge

In this study, we focus on three combinations: GPA template Procrustes, PCA template ridge regression, and pairwise optimal transport. These naming conventions are used throughout the paper.

## 3 Results

### 3.1 Using the same participants for template generation and alignment increases classification accuracy

Participants were grouped into two cohorts: a template generation cohort and an alignment cohort. A template fMRI response was derived by combining the movie-viewing fMRI responses of the template generation cohort. For each participant in the alignment cohort, a linear transformation was calculated mapping from their vertices to the template’s vertices, based on their movie viewing fMRI response. The task-independence and generalizability of these mappings was tested by using them to transform activation maps from the same participants during new tasks (Fig. 2). The similarity of participants’ activation maps was tested by training a linear classifier to decode task labels on the basis of task fMRI contrast maps as input. In all scenarios (with or without functional alignment), classification accuracy was well above chance. Chance level decoding accuracy was 1/18 (approximately 5.6%), as there were 18 possible task labels.

**Fig. 3.**
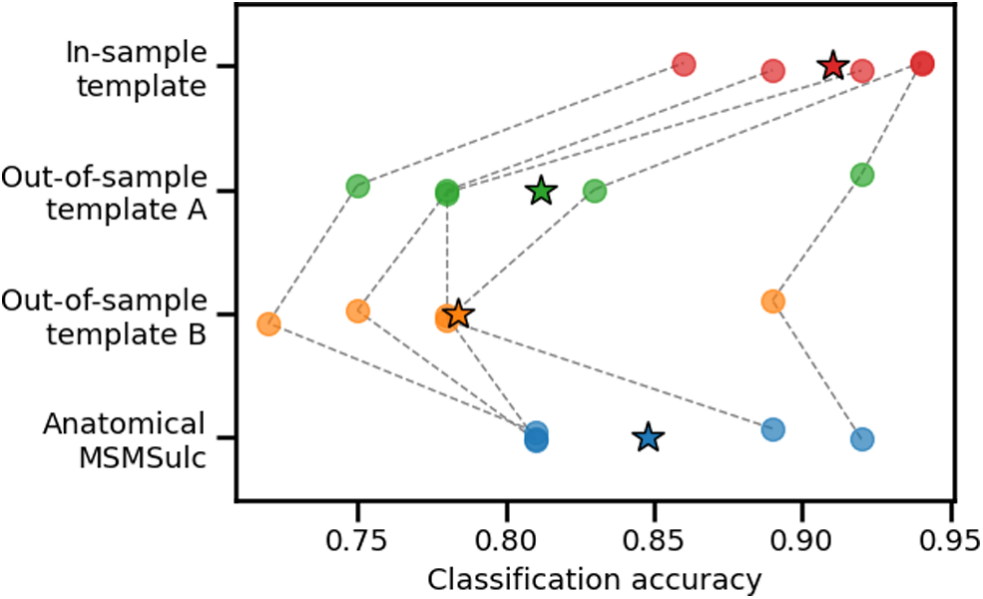
Out-of-sample versus in-sample template generation. Participants are numbered by their order in the HCP data release: 1, 2, etc. The movie-viewing responses of alignment participants 1–10 were aligned to a common template. Generalized Procrustes analysis was used to generate the template, combining movie-viewing data from participants 1–10 (in-sample template), 11–20, or 21–30 (out-of-sample templates A and B). Alignment transformations mapping from participants 1–10 to the template were used to transform their task fMRI responses to a common functional space. A support vector classifier was trained to classify task labels from task fMRI responses using 5-fold cross-validation. Each dot represents a single fold. Dashed lines connect the same data fold under different conditions. Mean values are indicated by a star. The “anatomical” row shows classification accuracy without any functional alignment.

Previous studies used the same participants for template generation and alignment, so that the template was “in-sample.” We tested whether generating a template with a different set of participants impacted the success of functional alignment. Template generation with an out-of-sample set of participants reduced subsequent task classification accuracy in the alignment cohort (Fig. 3, out-of-sample template A vs. in-sample, paired t-test, T(4) = 4.816, p = 0.009). The effect was replicated using a different set of participants for the out-of-sample template (Fig. 3, out-of-sample template B vs. in-sample, T(4) = 5.837, p = 0.004). The effect was replicated using a larger independent cohort (n = 20) of alignment and template generation participants (Supplementary Fig. 1), and also using data aligned with MSMAll instead of MSMSulc (Supplementary Fig. 2).Figure 3suggests that some variability in results can be expected depending on the specific participants used for template generation. This variability was not reduced by generating the template with more participants (Supplementary Fig. 3).

**Fig. 4.**
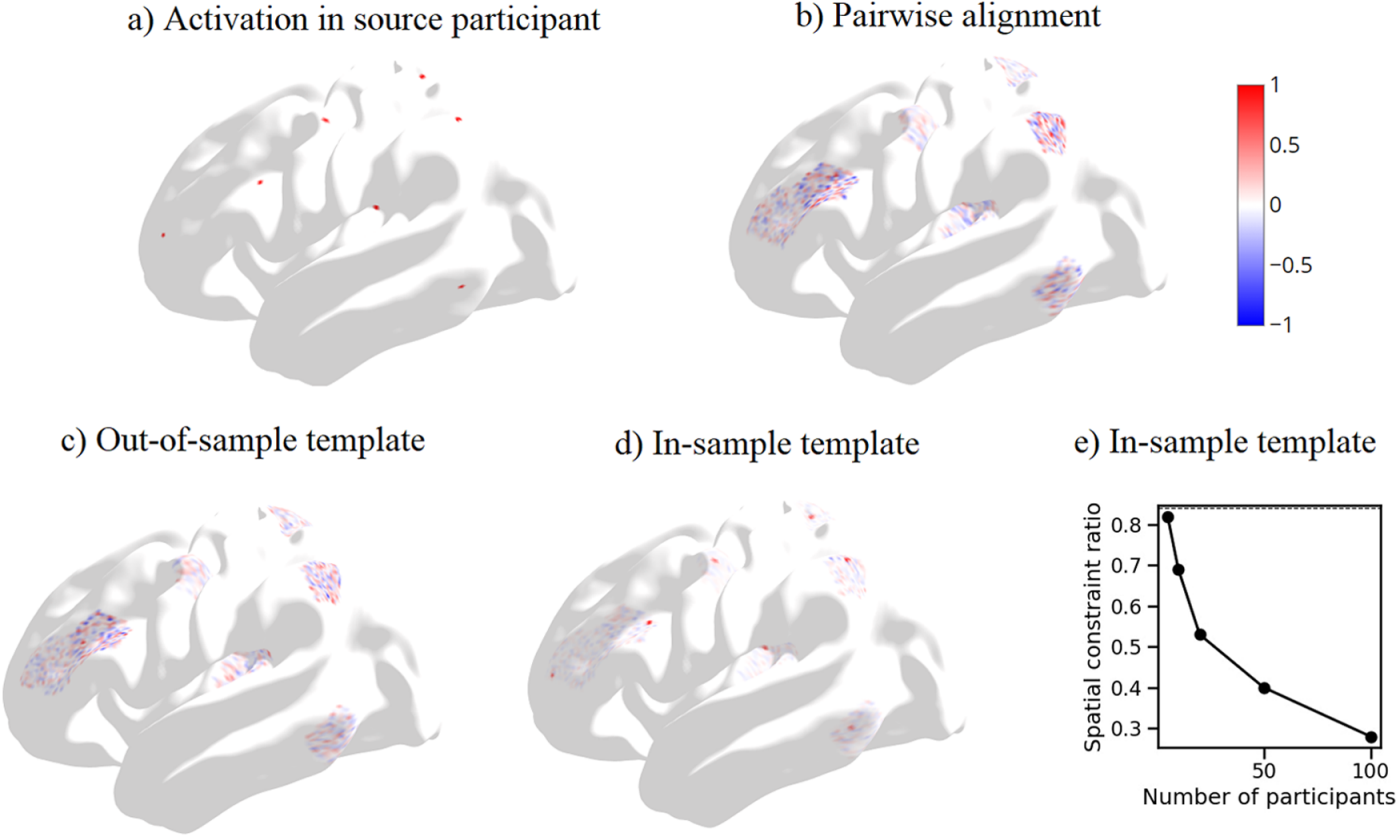
Visualizing the effect of template alignment on small localized functional activations. Alignment transformations were calculated to align movie viewing fMRI responses. Activations were visualized on the inflated HCP cortical surface. (a) Toy example of localized bump activations in a source participant (participant 1). Bump centers were randomly selected from the set of cortical vertices. Vertices within 1 mm of a bump center were set to value 1. All other vertices were set to value 0. (b) Bump activations in (a) were transformed using the pairwise alignment mapping from source participant to a single target participant. Spatial constraint ratio = 0.10. (c) Bump activations in (a) were transformed from the source participant’s space to template space. When the template was generated from participants 11–20 (out-of-sample template), activation magnitude was spread across whole parcels. Spatial constraint ratio = 0.12. (d) When the template was generated from participants 1–10 (in-sample templates), a majority of the activation magnitude remained within the original bumps. Spatial constraint ratio = 0.69. (e) Spatial constraint ratio with in-sample templates generated from 5, 10, 20, 50, and 100 participants.

We then visualized how the alignment transformation differed depending on whether a source participant was aligned to an in-sample or out-of-sample template. Note that in this case, the source participant’s data contribute to an in-sample template, but do not contribute to an out-of-sample template. We visualized how the functional alignment matrix, using the movie fMRI data, transformed a toy set of localized “bump-like” activations in a source participant (Fig. 4a).

The transformation from source participant to an in-sample template was spatially constrained, and the bulk of activation remained within the original bump (Fig. 4c). The mapping to an out-of-sample template transformed small bumps into diffuse activations and appeared similar to the mapping induced by pairwise alignment (Fig. 4b). We quantified the degree to which activations remained within the original as the proportion of the transformed activation’s Frobenius norm that was within the original bump,

$$S=\frac{{\left\|T\;\text{within original bump region}\right\|}_{F}}{{\left\|T\right\|}_{F}},$$

Where S is the spatial constraint ratio, T is the functional activation map after transformation to the template space, and${\left\|T\right\|}_{F}$refers to the Frobenius norm of T.

This spatial constraint ratio was markedly greater when the transformation mapped to an in-sample template. Similar but weaker effects were seen when the template was generated using the hyperalignment method or principal components analysis method instead of generalized Procrustes analysis (Supplementary Fig. 4).

### 3.2 In-sample templates bias towards anatomical alignment

We hypothesized the following explanation for the improved performance of within-sample alignment. Using the same participants for template generation and alignment testing produces a spatially constrained alignment matrix. Specifically, when a participant’s data contributes to the template, the participant’s time series at each vertex is likely to be correlated with the template’s time series at the same vertex, particularly when the template is derived from a small number of participants. The resulting participant-to-template transformation is more likely to map vertices in the participant to the same vertex in the template, and hence to have large positive values on the diagonal of the square matrix. This biases the transformation towards being an identity matrix, that is, anatomical alignment alone. When the source or target image is noisy, this implicit bias towards anatomical alignment improves interparticipant decoding accuracy for unrelated tasks.

This explanation suggests that the degree of spatial constraint is greatest with small sample sizes. Accordingly, at large sample sizes, each participant’s contribution to the template will be diluted, so the bias towards an identity matrix will also be attenuated. We confirmed this using template generated with different numbers of participants (Fig. 4e). At larger sample sizes, localized bump activations were transformed into activations spread more diffusely across the entire parcel. Hence, to avoid a spatial constraint that depends on sample size, different cohorts should be used for template generation and alignment.

### 3.3 Explicit integration of anatomical and functional information improves alignment

We then considered whether, even when different cohorts are used for template generation and alignment, alignment could be improved by explicitly adding anatomical information. Instead of mapping from a source participant to a template, we mapped from a source participant to a weighted average of source’s and template’s functional responses. Let$F_{X}^{v\times p}$be the functional response of participant X to movie-viewing, with v vertices and p timepoints. Let$F_{T}$be the template’s response. We generated a new target functional response,

$$F\prime_{T}=\gamma F_{X}+\left(1-\gamma \right)F_{T}$$

Alignment then finds matrix$R^{v\times v}$to minimize the difference between$R\times F_{X}$and$F\prime_{T}$. The parameterγis the proportion of the participant’s own data in the new template and varies from 0 (pure functional alignment) to 1. Withγ=1, the alignment maps from an image to itself. In the case of the Procrustes or optimal transport algorithms, this produces an identity transformation. As we used data registered with the MSMSulc algorithm, the identity transformation was equivalent to anatomical alignment alone without functional alignment. This technique effectively interpolates between purely anatomical and purely functional alignment.

**Fig. 5.**
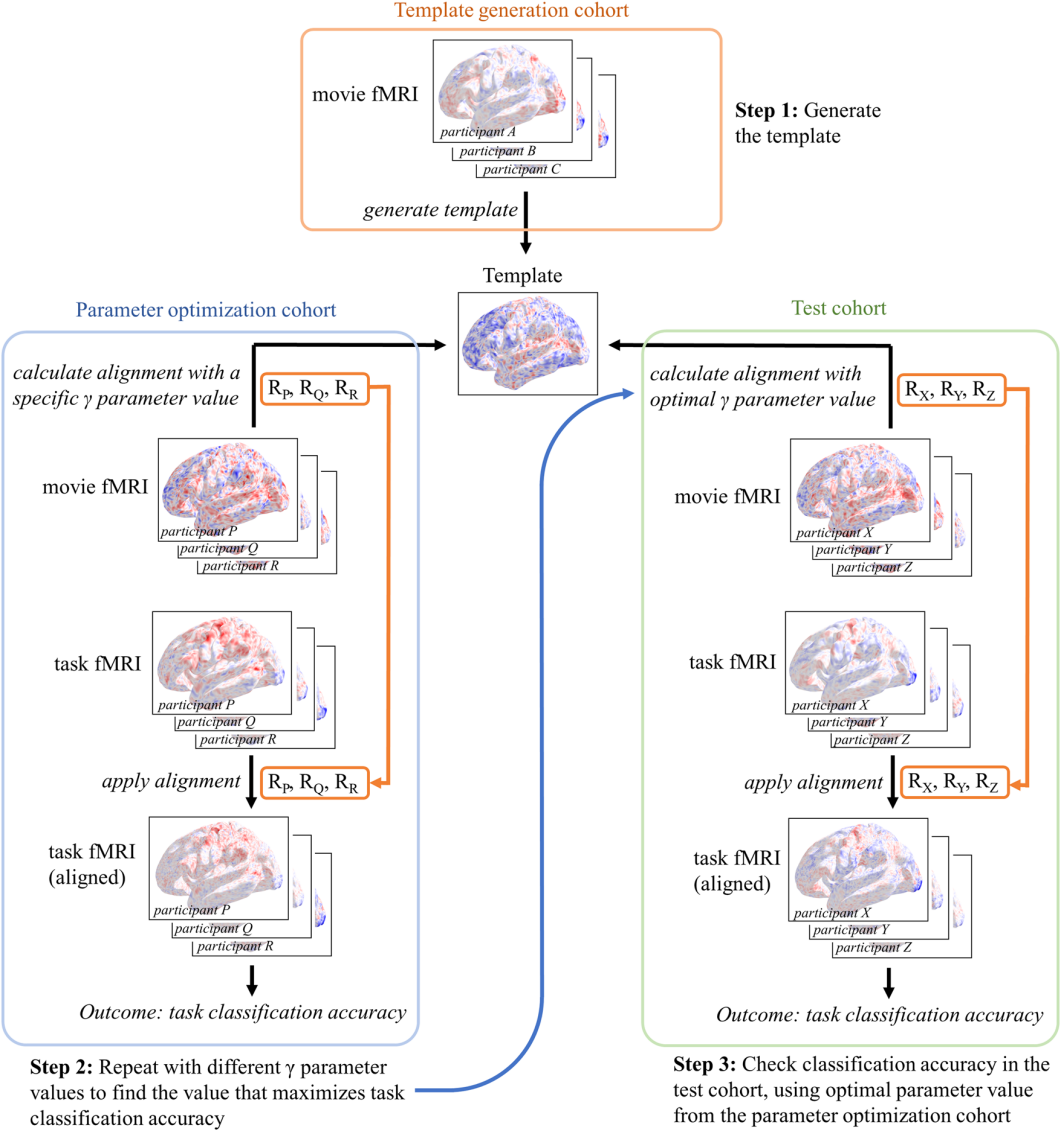
Schematic demonstrating the separation of cohorts. The template was generated with movie-viewing fMRI data from participants 21–40. The optimal parameter value was determined in participants 1–20. This parameter value was validated in three test cohorts, participants 41–60, 61–80, and 81–100.

**Fig. 6.**
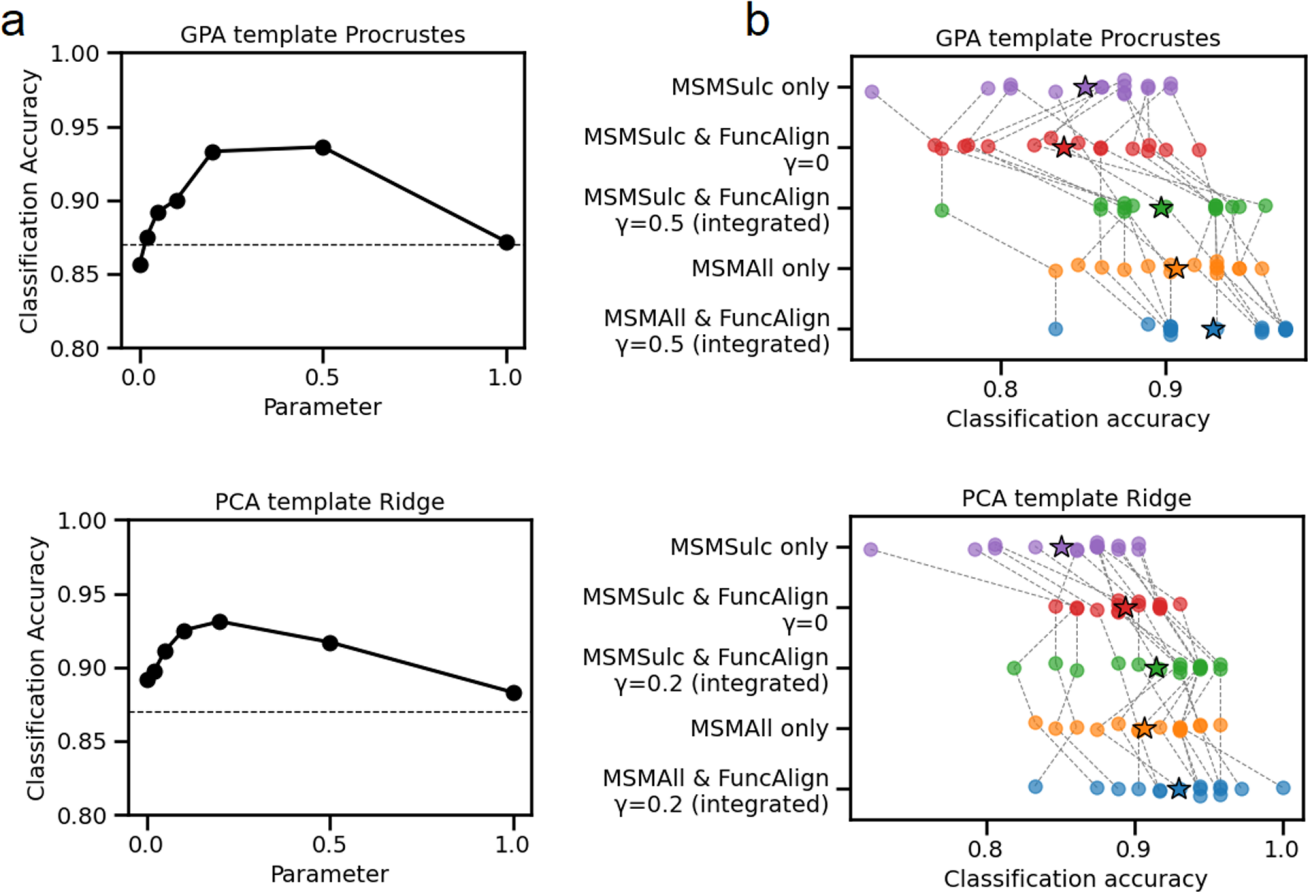
Integrating anatomical and functional information for alignment improves decoding accuracy for the GPA template Procrustes method (top) and the PCA template ridge regression (bottom). The template was generated with participants 21–40. (a) Task classification accuracy in the parameter optimization cohort (participants 1–20) as a function of parameter γ. γ = 0 corresponds to pure functional alignment, while greater values correspond to the inclusion of anatomical constraints. The horizontal dotted line indicates the value with anatomical alignment alone. (b) Task classification accuracy in test cohorts, with five different conditions: (1) anatomical alignment with MSMSulc, (2) MSMSulc followed by pure functional alignment, (3) MSMSulc followed by integrated alignment using optimal γ parameter from the parameter optimization cohort, (4) multimodal registration with MSMAll, and (5) MSMAll followed by integrated alignment. Each dot represents mean accuracy in a single fold, averaged across the participants within the fold and across the different task labels. Dashed lines connect the same data fold under different conditions. Mean values are indicated by a star. Integrated alignment significantly improved classification accuracies in the test set, for the GPA template Procrustes method and the PCA template ridge regression method.

We tested whether an intermediate parameter value (integrating MSMSulc anatomical and functional alignment) would improve alignment and classification compared to pure anatomical (MSMSulc only) or pure functional alignment (MSMSulc followed by functional alignment withγ=0). We also tested whether the optimal parameter value generalized across to a different cohort. Participants were divided into 3 cohorts, forγparameter optimization (participants 1–20), template generation (participants 21–40), and test (participants 41–60, 61–80, and 81–100) (Fig. 5). The template was generated with movie-viewing fMRI from the template generation cohort (participants 21–40) using the GPA method. For a given parameter value, Procrustes transformations were calculated from movie-viewing fMRI, mapping from each participant in the parameter optimization cohort (participants 1–20) to the template. These transformations were applied to the same participants’ task fMRI. The outcome measure was mean task classification accuracy in the parameter optimization cohort using 5-fold cross-validation. This procedure was repeated for different values of the parameterγspaced approximately logarithmically apart: 0, 0.02, 0.05, 0.1, 0.2, 0.5, and 1.0. Mean classification accuracy was maximized atγ=0.5(93.6%), with accuracy decreasing monotonically on either side to 85.6% with pure functional alignment (γ=0) and 87.2% with pure anatomical alignment (γ=1.0) (Fig. 6a).

We then evaluated whether the optimal γ value generalized to the test cohort. We tested whetherγ=0.5improved classification accuracy in each of the test cohorts (participants 41–60, 61–80, 81–100). Using movie-viewing fMRI, transformations were calculated aligning test cohort participants to the template generated from the template generation cohort (participants 21–40), withγ=0(pure functional alignment) orγ=0.5(integrated alignment). Transformations were applied to the same test cohort participants’ task fMRI data. Two-sided paired t-tests were used to compare conditions.

For clarity, in the following we specify whether the underlying data (template generation, parameter optimization, and test cohorts) were registered with MSMSulc or MSMAll. Integrated alignment (MSMSulc & FuncAlignγ=0.5) yielded significantly greater classification accuracy than pure functional alignment (MSMSulc & FuncAlignγ=0) (T(14) = 5.492, p < 0.001) and pure anatomical registration (MSMSulc only) (T(14) = 6.665, p < 0.001) (Fig. 6b). We then compared integrated alignment to MSMAll. MSMAll calculates a diffeomorphic registration based on multimodal data, including resting-state connectivity. Functional alignment can be seen as an optional non-diffeomorphic alignment step, conducted on fMRI data pre-aligned with either MSMSulc or MSMAll. With MSMSulc, integrated alignment interpolates between anatomical and functional alignment. With MSMAll-aligned data, integrated alignment mixes MSMAll and functional alignment. Integrated alignment (MSMSulc & FuncAlign*γ=0.5*) was not significantly different from MSMAll alone (T(14) = -1.427, p = 0.175). Finally, we queried whether non-diffeomorphic integrated alignment produced additional benefit on data pre-aligned with MSMAll. Integrated alignment (MSMAll & FuncAlignγ=0.5) yielded significantly greater accuracy than MSMAll alone (T(14) = 5.242, p < 0.001).

Results were similar even when a different functional alignment algorithm was used. With the PCA template ridge regression method, classification accuracy in the parameter optimization set was maximized withγ=0.2. In the test set, integrated alignment withγ=0.2yielded significantly greater accuracy than both pure functional alignment withγ=0(T(14) = 3.328, p = 0.005), and pure anatomical registration with MSMSulc (T(14) = 8.463, p < 0.001). Integrated alignment (MSMSulc & FuncAlignγ=0.2) was not significantly different from MSMAll (T(14) = 1.305, p = 0.213). However, the combination of MSMAll pre-alignment and subsequent integrated alignment had greater accuracy than MSMAll alone (T(14) = 3.719, p = 0.002) (Fig. 6, bottom). All significant results above remained significant even if the non-parametric Wilcoxon signed-rank test was used instead of the paired t-test (Supplementary Tables 1 and 2).

**Fig. 7.**
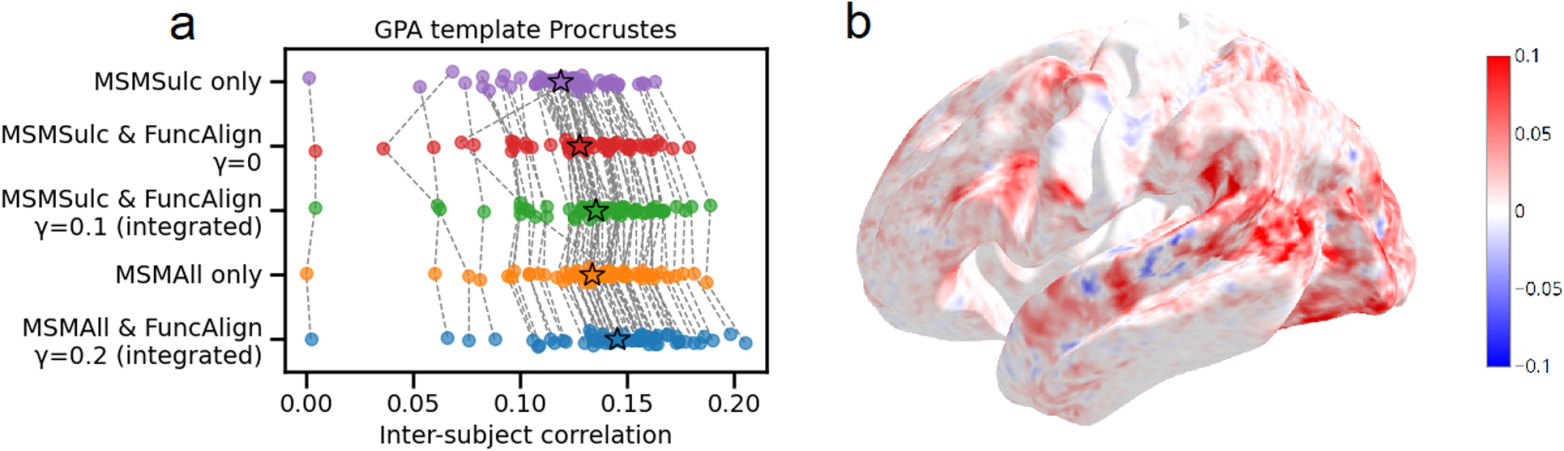
Integrating anatomical and functional information improves inter-subject correlation (ISC). The template was generated with participants 21–40. The parameter γ was optimized for ISC in participants 1–20. (a) ISC in test cohorts. Each dot represents ISC between a single participant and all other participants. Dashed lines connect the same participant. (b) Vertex-wise ISC difference between MSMAll only, and MSMAll followed by integrated alignment (γ = 0.2). Positive values indicate superiority of adding integrated alignment. Correlation values were truncated to [-0.1, 0.1] to improve color visualization.

To assess whether results depended on validation modality (classification accuracy in task fMRI), we also validated with inter-subject correlation in movie-viewing fMRI. Alignment transformations from the test cohort to the template were calculated using movie-viewing runs 1 and 2. These transformations were applied to the validation data, movie runs 3 and 4 in the test cohort. The overlap between participants’ transformed data was measured with inter-subject correlation (ISC) instead of classification accuracy. For each vertex in each participant, we calculated the Pearson correlation between the participant’s time series and all other participants’ time series. ISCs were averaged across vertices, yielding a single value for each participant. ISC was significantly greater with integrated alignment compared to pure anatomical (MSMSulc) (T(59) = 18.468, p < 0.001) and pure functional alignment (T(59) = 12.533, p < 0.001). Integrated alignment applied to MSMSulc data yielded greater ISC than MSMAll alone (T(59) = 2.041, p = 0.046). Integrated alignment applied to MSMAll data also improved on MSMAll alone (T(59) = 22.526, p < 0.001). Integrated alignment improved on MSMAll in cortical areas, including occipital, temporal, and fronto-parietal regions (Fig. 7).

**Fig. 8.**
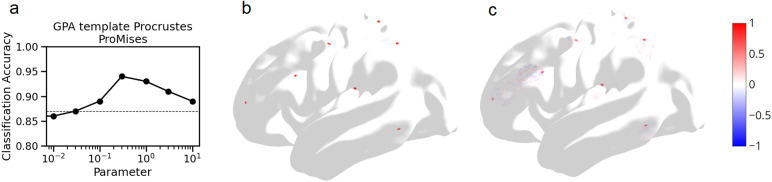
(a) Task fMRI classification accuracy in 20 participants using GPA template Procrustes alignment with the ProMises spatial regularization, as a function of regularization parameter k. Parameter values used were [0, 0.01,0.03,0.1,0.3,1,3,10]. k = 0 corresponds to no regularization. The horizontal dotted line indicates the value with anatomical alignment alone. (b) Toy example of localized bump activations in a source participant. (c) Bump activations from (b) were transformed by the ProMises model regularized transformation (k = 0.3) mapping from the source participant to the template. The resulting activations in template space are shown here.

We then compared our technique with the ProMises model. This approach penalizes mappings between spatially distant vertices by introducing the geodesic distance between vertices, multiplied by regularization parameter k, to the Procrustes equation (seeSupplementary Methods 4for details) (Andreella et al., 2022). The ProMises model cannot be used with other source-to-target algorithms like ridge regression or optimal transport. Therefore, we compared the ProMises model to our results for the GPA template Procrustes method (Fig. 6top) using the same template generation, parameter optimization, and test cohorts. In the parameter optimization cohort, classification accuracy was optimized with k = 0.3. The ProMises model produced a similar spatial constraint to our method (Fig. 8). In the test cohort, we did not find a significant difference between the ProMises model and our integrated alignment, irrespective of whether the underlying data were registered with MSMSulc (T(14) = -1.602 p = 0.131) or MSMAll (T(14) = -0.292, p = 0.774).

Next, we asked whether integrating anatomical and functional alignment could improve pairwise alignment from one participant to another. For pairwise alignment, the alignment target F_T_refers to the target participant rather than a template. Pairwise alignment does not have any implicit spatial constraint, unlike the situation with template alignment when the same participants are used for template generation and alignment. Our integrated alignment improved classification accuracy in pairwise alignment, irrespective of whether the source-to-target algorithm was Procrustes or optimal transport (Supplementary Fig. 5).

## 4 Discussion

Our study of template alignment emphasizes the importance of using separate cohorts for template generation and template testing. Using the same cohorts for template generation and alignment biases the alignment transformation towards identity, particularly for the smaller sample sizes used in most functional alignment studies. This process effectively interpolates between pure functional alignment, which is sensitive to noise, and standard anatomical alignment. This may also explain why template alignment typically outperforms pairwise alignment.

In machine learning, separating the training and test cohorts increases the generalizability of predictions. In functional alignment, separating the cohorts ensures the generalizability of the template to other cohorts. Mixing the cohorts also limits generalizability of the overall result to larger samples. This is because smaller studies have a greater bias towards pure anatomical alignment, and their results will tend not to generalize to larger samples. Therefore, we recommend that templates should be generated using a separate cohort.

These findings motivated the explicit incorporation of anatomical constraints into functional alignment. Standard functional alignment does not involve spatial constraints beyond limiting mappings to be within a single parcel or a “searchlight.” Our method introduces a balance between anatomical (MSMSulc) and functional alignment, with the parameter optimization procedure ensuring that functional data are appropriately weighted. This approach is particularly beneficial in scenarios where functional data may be noisy, as it downweighs such data relative to alignment using anatomical landmarks. We noted that the optimal balance of anatomical and functional alignment also differs depending on the alignment technique. We selected the optimal parameter through an approximately logarithmic grid search, but users could alternatively use iterative methods to converge on the optimal value. Users should establish the efficacy of integrated alignment in a parameter optimization subset of their data before application in a test set. For example, if the plot of accuracy versus parameter value has multiple shallow local maxima without a substantial accuracy improvement, then simpler approaches like pure functional or anatomical alignment could be used in the test cohort. Due to the reliance on a relatively small number of template participants, we recommend that templates are generated from 10 or more participants with low head motion.

Our method extends the hyperalignment method (Haxby et al., 2011) and uses non-diffeomorphic transformation to align individuals to a functional space. The method differs from diffeomorphic approaches for incorporating functional data into interparticipant alignment like MSMAll, in that it permits discontinuous transformations that do not preserve local topology. Diffeomorphic methods, even when using functional data (Conroy et al., 2013;Sabuncu et al., 2010), cannot fully address idiosyncrasies in functional topography. Integrated alignment outperformed pure anatomical registration (MSMSulc) but was not significantly different from MSMAll. This highlights the need to compare functional alignment against modern diffeomorphic approaches. The addition of integrated alignment to MSMAll registration improved performance by a modest, statistically significant margin. When the original data are registered with MSMSulc, our approach interpolates between functional alignment and anatomical registration. When the original data are registered with MSMAll, integrated alignment is reconceptualized as interpolating between diffeomorphic and non-diffeomorphic approaches. While MSMAll+ FuncAlign had superior performance, this pipeline is not readily transferrable to volume-based data because there is no direct volumetric analogue of MSMAll. In contrast, the MSMSulc+ FuncAlign pipeline can be translated to volume-based data with registration to MNI space replacing MSMSulc.

Our method also differs from various non-spatial approaches for regularizing functional alignment. Techniques such as ridge regression or entropic regularization in optimal transport target the sparsity of the transformation, rather than constraining the mapping based on anatomical landmarks (Bazeille et al., 2019). The approach introduced here is similar to the ProMises model in spatially constraining alignment and improving classification accuracy (Fig. 8). However, our method is conceptually simpler, and can be used with all functional alignment algorithms unlike the ProMises model which is situated within the Procrustes algorithm.

A particular issue to note with the present method is that it penalizes the mapping from vertex i in participant X to vertex j in participant Y if i ≠ j, irrespective of the distance between vertices i and j. That is, the distance between vertices i and j is not explicitly considered. However, adjacent vertices will tend to have similar time series due to the intrinsic spatial smoothness of fMRI data. Hence, our method is implicitly more likely to map vertices to nearby vertices than to distant vertices due to data smoothness alone. This allows the width of the spatial constraint to adapt based on local spatial smoothness, which could be advantageous over regularization methods employing a uniform spatial width kernel across all brain regions.

We used the Schaefer parcellation with 300 parcels (Kong et al., 2021;Schaefer et al., 2018). Functional alignment is relatively insensitive to parcellation choice. Parcellation only constrains which vertices in the source participant are allowed to match which vertices in the target participant. As long as parcels are larger than the residual misalignment after MSMSulc or MSMAll, parcellation choice has minimal impact. Previous work has shown similar alignment performance using Schaefer parcellations between 100 and 1000 parcels, and parcellations directly derived from the functional data by k-means or hierarchical k-means clustering (Bazeille et al., 2021). Searchlight hyperalignment (Guntupalli et al., 2016), which sidesteps the problem of parcellation choice by using multiple overlapping circular parcels, imposes a greater computational burden without concomitant performance gain (Bazeille et al., 2021). We did not use individualized parcellations, because the alignment methods used required source and target spaces to contain the same number of vertices. Individualized parcellations yield different parcel sizes in different individuals. Future work could test integrated alignment with functional parcellations derived from our movie viewing data, or individualized parcellations in conjunction with alignment methods for unbalanced datasets.

The method also differs from Fused Unbalanced Gromov Wasserstein alignment (FUGW), situated within the framework of optimal transport (Thual et al., 2023). FUGW includes areal regularization by penalizing transformations that change the distance between vertex pairs, but discards prior information from MSMSulc or MSMAll. Integrated alignment provides spatial regularization by penalizing single vertices moving to distant anatomical loci. Consequently, it requires pre-alignment with MSMSulc or MSMAll. The Procrustes algorithm used in our analyses adds further regularization, constraining vector norms of brain activation maps to be unchanged by the transformation. When aligning across species in participant-specific native spaces, source and target participants’ data exist on different anatomical meshes. In this context, FUGW is more appropriate. However, when anatomical information is present, incorporating this information with integrated alignment is beneficial. Future work could combine these two approaches. This would require three separate regularization terms—for the anatomical prior (as in integrated alignment), to preserve distance relationships between vertices, and to permit unbalanced alignments (as in FUGW).

The method introduced in this study is situated within a larger class of alignment approaches that leverage the strengths of both anatomical and functional landmarks, accounting for individual variations in brain function while maintaining a consistent anatomical reference. Anatomical alignment methods remain dominant due to concerns that functional alignment introduces noise by overfitting to noisy functional data. Balanced approaches which retain anatomical constraints in the absence of reliable functional landmarks may allay such concerns. These methods increase the scope for precision brain mapping by capturing individual differences in fine-scale brain function, improving our ability to predict and classify behavior from neuroimaging.

## Supplementary Material

Supplementary Material

## Data Availability

Code to perform analyses is available at our fork of the*fmralign*package:https://github.com/jaysonjeg/fmralign Data were from the Human Connectome Project and can be accessed athttps://db.humanconnectome.org/
