# Supplementary material for: Integrating anatomical and functional landmarks for interparticipant alignment of imaging data
Source: Imaging Neurosci (Camb). 2024 Aug 1;2:imag-2-00253. doi: 10.1162/imag_a_00253 (PMC12272198; doi:10.1162/imag_a_00253)
Supplement: Supplementary Material [file imag_a_00253-supp.pdf]

Supplementary materials

Supplementary Methods 1. Human Connectome Project data details

7T fMRI movie viewing – 4 runs x 15 mins = 1 hour. Time series were minimally preprocessed (Glasser et al., 2013) and cleaned with ICA-FIX. In each run, participants viewed several videos separated by the word "REST" appearing on screen. Timepoints containing the word "REST" were removed from the time series. However, the first 10 seconds after the end of each video was retained, as this segment contained the haemodynamic response to the video. The first 10 seconds of each video was removed for the same reason. For each run, each vertex's time series was standardized to zero mean and unit variance. The 4 runs were then concatenated.

3T fMRI tasks –Contrast maps during 7 tasks. Each task had multiple basic contrasts, for a total of 18 contrast maps. Each map was a participants' average across trials during the task. The tasks (and contrasts) used were motor (LF, LH, RF, RH), working memory (2BK_BODY, 2BK_FACE, 2BK_PLACE, 2BK_TOOL), emotion (FACES, SHAPES), gambling (PUNISH, REWARD), language (MATH, STORY), relational (MATCH, REL), and social (RANDOM, TOM).

Supplementary Methods 2. Outcome measures for assessing functional alignment:

Functional alignment aims to improve the concordance between participants' brain maps to the same stimulus, but maintain the distinguishability of participants' responses to different stimuli. Correlative measures and interparticipant classification accuracy are two possible outcome measures for assessing functional alignment. Correlative measures include intersubject correlation (ISC) (Jiahui et al., 2023), or the correlation between a participant's actual and predicted fMRI maps. Correlative measures are more sensitive to low-level characteristics like smoothing. Changes in correlative measures after functional alignment can arise due to image processing effects (Bazeille et al., 2021). In contrast, interparticipant classification accuracy provides a more direct measure of the concordance of participants' responses to the same stimulus and distinguishability of their responses to different stimuli

Interparticipant classification accuracy also has some limitations as an unbiased comparator of alignment methods. Functional alignment methods, by recombining values within a parcel, can destroy information that is used by a classifier. One example is the mean activation across a parcel. Given an activation brain map divided into 300 parcels, consider the set of 300 within-parcel mean activations. Since low spatial frequencies account for a majority of variation across any given brain map, these 300 within-parcel means may be sufficient to accurately distinguish between different brain maps. With anatomical alignment, the classifier has access to these within-parcel means. To demonstrate the importance of within-parcel means, for each task fMRI map, we subtracted from each vertex, the mean value of the parcel that it was contained within. In anatomically aligned data, this parcel-mean normalization reduced classification accuracy from 84% to 69%.

Some functional alignment methods destroy within-parcel means. For example, Procrustes alignment and ridge regression do not preserve within-parcel means, while optimal transport and permutation do. Therefore, it is possible that Procrustes alignment improves within-parcel correlations between participants' brain maps, but underperforms on classification tasks simply because it has destroyed within-parcel means. To address this, given a task fMRI brain map, the 300 within-parcel means were calculated before functional alignment. After the map was transformed by functional alignment, the within-parcel means were restored. Each transformed brain map was thus extended from a vector of 59,412 vertices, to a vector of 59,412 vertices + 300 within-parcel summary measures. These appended maps were used for interparticipant decoding, to make the comparison between alignment methods fairer. We tested whether appended maps improved classification accuracy. 20 participants were aligned to a template derived from 20 other participants. For the Procrustes alignment method, appended maps had significantly greater classification accuracy (85.6%) than non-appended maps (83.1%) (paired t-test across folds, $T(4)=2.741$, $p=0.026$). We saw similar improvement with 40 participants ($T(4)=6.517$, $p=0.001$) and when using the PCA ridge regression method ($T(4)=4.795$, $p=0.004$). We appended parcel-specific means after functional alignment to make the comparison between standard alignment (MSMSulc, MSMAll) and functional alignment fairer, acknowledging that this is only an approximate solution to restoring some of the information lost through functional alignment.

Supplementary Methods 3. Template generation methods

Supplementary Methods 3.1: Template generation with Generalized Procrustes analysis

In the following pseudocode, rescaling refers to multiplying an image $F^{v \times p}$ by a scalar so that its Frobenius norm becomes the average Frobenius norm of all participants' images before alignment. By default we used nIterations=1 in keeping with the *fmralign* package default.

```
FOR EACH image IN participantImages DO
  image <- Rescale(image)
END FOR

template <- Mean(participantImages)

FOR i FROM 1 TO nIterations DO
  template <- Rescale(template)
  alignedRescaledImages <- EMPTY LIST
  FOR EACH image IN participantImages DO
    transformation <- CalculateAlignment (image, template)
    alignedRescaledImage <- Rescale(ApplyTransformation(image, transformation))
    APPEND alignedRescaledImage TO alignedRescaledImages
  END FOR
```

```
    template <- Mean(alignedRescaledImages)
  END FOR

  template <- Rescale(template)
  RETURN template
```

Supplementary Methods 3.2: Template generation with hyperalignment

In the following pseudocode, z-scoring refers to z-scoring along rows (across timepoints, within vertices) of image $F^{v \times p}$.

```
// Level 1
Level1AlignedImages <- EMPTY LIST
target <- firstParticipantImage
APPEND target TO Level1AlignedImages
target <- ZScore(target)

FOR EACH image IN remainingParticipantImages DO
  transformation <- CalculateAlignmentToTarget(image, target)
  alignedZScoredImage <- ZScore(ApplyTransformation(image, transformation))
  APPEND alignedZScoredImage TO Level1AlignedImages
  target <- Zscore(Mean(target, alignedZScoredImage))
END FOR

// Level 2
Level2AlignedImages <- EMPTY LIST
FOR EACH image IN participantImages DO
  otherParticipantsImages <- Level1AlignedImages EXCLUDING image
  target <- Mean(otherParticipantsImages)
  transformation <- CalculateAlignmentToTarget(image, target)
  alignedZScoredImage <- ZScore(ApplyTransformation(image, transformation))
  APPEND alignedZScoredImage TO Level2AlignedImages
END FOR

template <- Zscore(Mean(Level2AlignedImages))
RETURN template
```

Supplementary Methods 3.3: Template generation with principal components analysis

Let $F_i^{v \times p}$ represent participant i's movie viewing response in a given parcel, where v is the number of vertices within that parcel. Participants' images for the given parcel were concatenated vertically (across vertices) to form matrix $F^{(v \times nParticipants) \times p} = [F_i ; F_j ; F_k ; \ldots]$. Dimensionality was reduced with PCA, where each principal

component P $^{(v \times nParticipants)}$ is a linear combination of vertices from multiple participants. The first v components were retained, and their time series were together yielded a template $F_{template}{}^{v \times p}$ where each "template vertex" was a linear combination of vertices from multiple participants.

The Procrustes method then rotated this template so that its "vertices" were better aligned to the group mean time series in anatomical space. A source image was constructed as identical copies of the template tiled horizontally: $S^{v \times (p \times nParticipants)} = [F_{template}{}^{v \times p}, F_{template}{}^{v \times p}, \ldots]$. The target image was all participants' original images concatenated horizontally (across timepoints): $T^{v \times (p \times nParticipants)} = [F_i, F_j, F_k, \ldots]$. A rotation was calculated mapping from this source image to the target image. This transformation was applied to the template $F_{template}{}^{v \times p}$. Finally, columns of the rotated template image were z-scored to yield the final template.

<u>Supplementary Methods 4. ProMises model</u>

Let $F_X{}^{v \times p}$ represent the movie viewing functional response of participant X, with v vertices and p timepoints. The standard Procrustes algorithm finds mapping $R^{v \times v}$ from F_X to F_Y as follows.

$U D V^T = SVD (F_Y{}^T F_X)$

$R = U V$

The ProMises model extends this algorithm by penalizing mappings between spatially distant vertices(Andreella et al., 2022). Let $d_{i,j}$ represent the geodesic distance between vertices i and j in the Human Connectome Project mid-thickness surface. Then matrix G with elements $G_{i,j} = \exp(-d_{i,j})$ is a symmetric matrix with ones on the diagonal.

$U D V^T = SVD(F_Y{}^T F_X + kG)$, where hyperparameter k determine the extent of regularization

$R = U V$

The original paper applied this regularization to the Procrustes algorithm with the hyperalignment template. We used this regularization with the GPA template.

<u>Supplementary results</u>

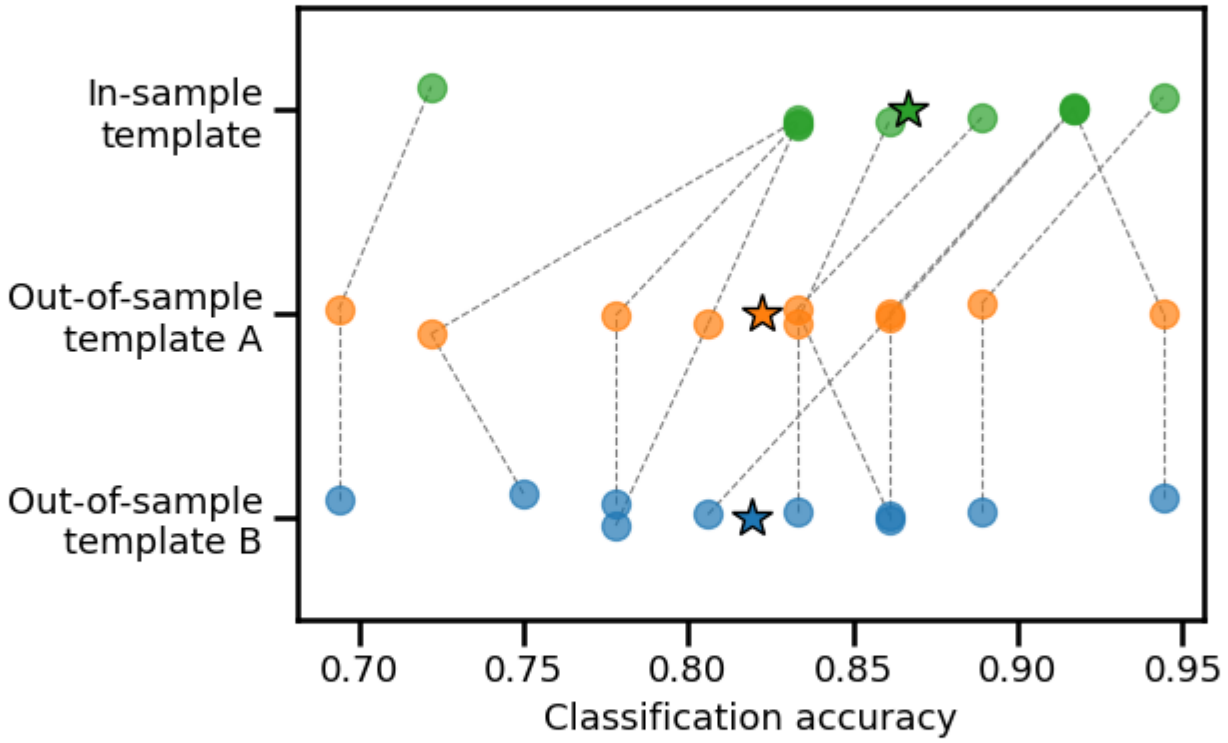

Supplementary Figure 1. Out-of-sample vs in-sample template generation with a larger sample size. Participants are numbered by their order in the HCP data release: 1, 2, etc. The movie-viewing responses of alignment participants 80-100 were aligned to a common template. Generalized Procrustes analysis was used to generate the template, combining movie-viewing data from participants 80-100 (in-sample template), 100-120 or 120-140 (out-of-sample templates A and B). Alignment transformations mapping from participants 80-100 to the template were used to transform their task fMRI responses to a common functional space. A support vector classifier was trained to classify task labels from task fMRI responses using 10-fold cross validation. Each dot represents a single fold. Dashed lines connect the same data fold under different conditions. Mean values are indicated by a star. Template generation with an out-of-sample set of participants reduced subsequent task classification accuracy in the alignment cohort (in-sample vs. out-of-sample template A, $T(9)=4.025$, $p=0.003$; in-sample template vs. out-of-sample template B, $T(9)=4.050$, $p=0.003$).

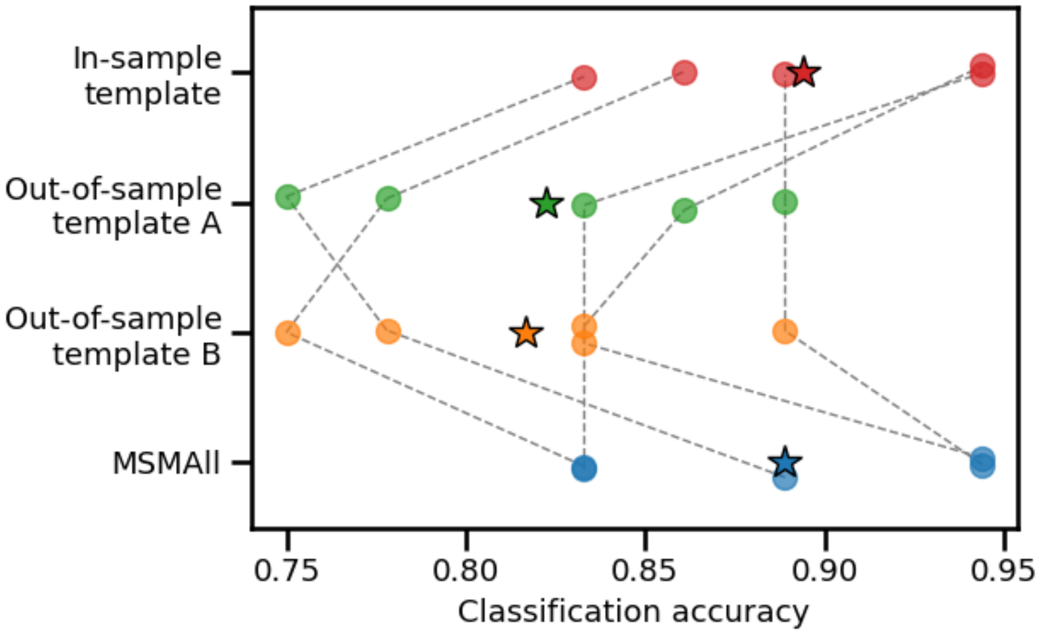

Supplementary Figure 2. Out-of-sample vs in-sample template generation using data aligned with MSMAll instead of MSMSulc. Movie-viewing responses of participants 0-10 were aligned using Generalized Procrustes analysis, to a template generated from participants 0-10 (in-sample template), 10-20 or 20-30 (out-of-sample template). Each dot represents classification accuracy from a single fold. Template generation with an out-of-sample set of participants reduced subsequent task classification accuracy (in-sample vs. out-of-sample template A, T(4)=3.830, p=0.019; in-sample template vs. out-of-sample template B, T(4)=3.492, p=0.025).

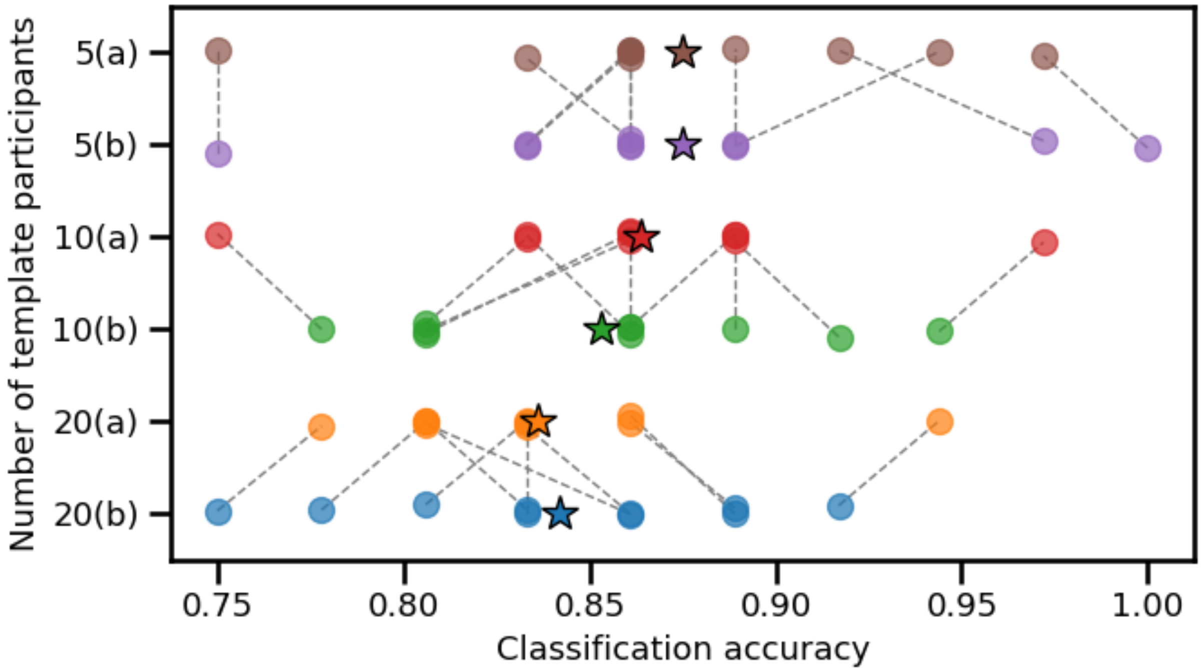

Supplementary Figure 3. Variability in results between different choices of template participants, as a function of the number of template participants. The movie-viewing responses of participants 1-20 were aligned to a common template using Generalized Procrustes analysis. Alignment transformations mapping from participants 1-20 to the template were used to transform their task fMRI response. A support vector

classifier was trained to classify task labels from task fMRI responses using 10-fold cross validation. Each dot represents a single fold. Each row represents a different choice of template participants, with variability in the number of template participants (5, 10, 20) and in the specific participants used (a, b). Dashed lines connect the same data fold under different choices of template participants (keeping the number of template participants constant).

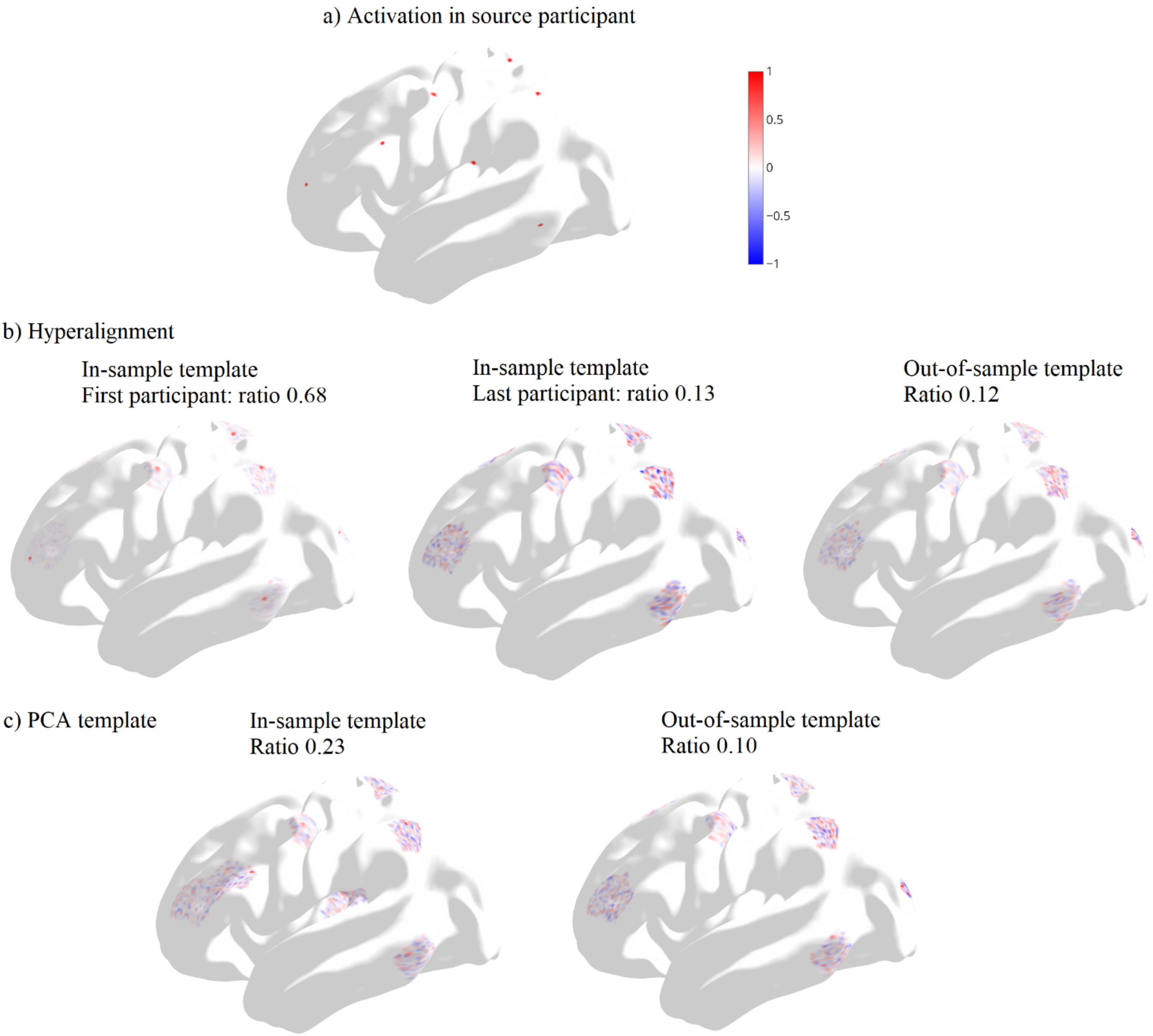

Supplementary Figure 4. Visualizing the effect of in-sample and out-of-sample templates on the spatial constraint ratio, using different methods of template generation. a) Toy example of localized bump activations in a source participant (participant 1). b) Bump activations in (a) were transformed to a template generated with hyperalignment. Since the hyperalignment method sequentially adds participants to a growing template, the template is very similar to the functional response of the first participant added. The images show, from left to right, the mapping from the first participant (1) to the template generated from participants 1-10, the mapping from the last participant (10) to the template generated from participants 1-10, and the mapping from

participant 1 to the template generated from participants 11-20 c) Bump activations in (a) were transformed to a template generated with principal components analysis. This method remixes vertices in different participants to extract low-dimensional components.

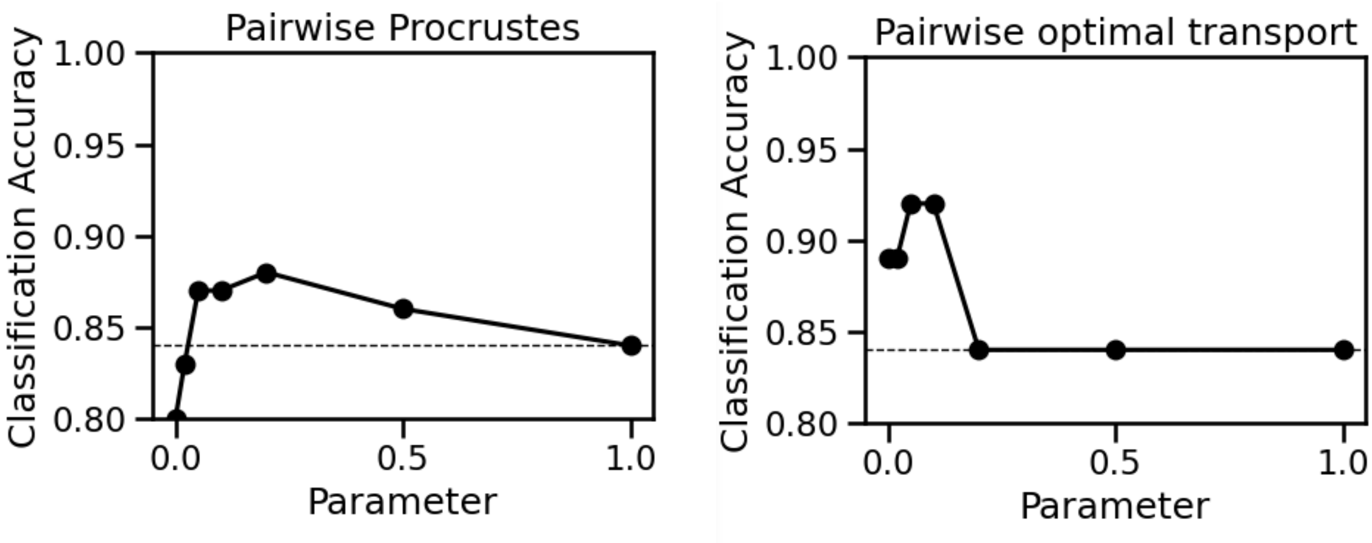

Supplementary Figure 5. Task fMRI classification accuracy in 10 participants using pairwise Procrustes alignment or pairwise optimal transport methods, as a function of the parameter γ. γ=0 corresponds to pure functional alignment, while greater values correspond to the inclusion of anatomical constraints. The horizontal dotted line indicates the value with anatomical alignment alone.

Condition	W-statistic	p-value
MSMSulc & FuncAlign ($\gamma = 0.5$) vs. MSMSulc & FuncAlign ($\gamma = 0$)	0	<0.001
MSMSulc & FuncAlign ($\gamma = 0.5$) vs. MSMSulc only	1	<0.001
MSMSulc & FuncAlign ($\gamma = 0.5$) vs. MSMAll only	31	0.177
MSMAll & FuncAlign ($\gamma = 0.5$) vs. MSMAll only	0	0.003

Supplementary Table 1. Comparison of mean classification accuracies with GPA template Procrustes, under pre-alignment (MSMSulc or MSMAll), followed by either no functional alignment, pure functional alignment ($\gamma = 0$), or integrated alignment (optimal γ). The per-fold classification accuracies for each condition are shown in Figure 6b. Wilcoxon signed-rank test was used to test for differences in paired ranks between conditions. Results with paired t-test are given in the main text.

Condition	W-statistic	p-value
MSMSulc & FuncAlign ($\gamma = 0.2$) vs. MSMSulc & FuncAlign ($\gamma = 0.5$)	10	0.023

MSMSulc & FuncAlign ($\gamma = 0.2$) vs. MSMSulc only	1	0.001
MSMSulc & FuncAlign ($\gamma = 0.2$) vs. MSMAll only	36	0.298
MSMAll & FuncAlign ($\gamma = 0.2$) vs. MSMAll only	8	0.009

Supplementary Table 2. Comparison of mean classification accuracies with PCA template Ridge, under pre-alignment (MSMSulc or MSMAll), followed by either no functional alignment, pure functional alignment ($\gamma = 0$), or integrated alignment (optimal γ). The per-fold classification accuracies for each condition are shown in Figure 6b. Wilcoxon signed-rank test was used to test for differences in paired ranks between conditions. Results with paired t-test are given in the main text.
